# Implementing Electronic Health Records in Primary Care Using the Theory of Change: Nigerian Case Study

**DOI:** 10.2196/33491

**Published:** 2022-08-11

**Authors:** Taiwo Adedeji, Hamish Fraser, Philip Scott

**Affiliations:** 1 School of Computing, University of Portsmouth Portsmouth United Kingdom; 2 Brown Center for Biomedical Informatics, Brown University Providence, RI United States; 3 Institute of Management and Health, University of Wales Trinity Saint David Carmarthen United Kingdom

**Keywords:** theory of change, electronic health records, maternal and child health, primary health center, success criteria

## Abstract

**Background:**

Digital health has been a tool of transformation for the delivery of health care services globally. An electronic health record (EHR) system can solve the bottleneck of paper documentation in health service delivery if it is successfully implemented, but poor implementation can lead to a waste of resources. The study of EHR system implementation in low- and middle-income countries (LMICs) is of particular interest to health stakeholders such as policy makers, funders, and care providers because of the efficiencies and evidence base that could result from the appropriate evaluation of such systems.

**Objective:**

We aimed to develop a theory of change (ToC) for the implementation of EHRs for maternal and child health care delivery in LMICs. The ToC is an outcomes-based approach that starts with the long-term goals and works backward to the inputs and mediating components required to achieve these goals for complex programs.

**Methods:**

We used the ToC approach for the whole implementation’s life cycle to guide the pilot study and identify the preconditions needed to realize the study’s long-term goal at Festac Primary Health Centre in Lagos, Nigeria. To evaluate the maturity of the implementation, we adapted previously defined success factors to supplement the ToC approach.

**Results:**

The initial ToC map showed that the long-term goal was an improved service delivery in primary care with the introduction of EHRs. The revised ToC revealed that the long-term change was the improved maternal and child health care delivery at Festac Primary Health Center using EHRs. We proposed a generic ToC map that implementers in LMICs can use to introduce an optimized EHR system, with assumptions about sustainability and other relevant factors. The outcomes from the critical success factors were sustainability: the sustained improvements included trained health care professionals, a change in mindset from using paper systems toward digital health transformation, and using the project’s laptops to collect aggregate data for the District Health Information System 2–based national health information management system; financial: we secured funding to procure IT equipment, including servers, laptops, and networking, but the initial cost of implementation was high, and funds mainly came from the funding partner; and organizational: the health professionals, especially the head of nursing and health information officers, showed significant commitment to adopting the EHR system, but certain physicians and midwives were unwilling to use the EHR system initially until they were persuaded or incentivized by the management.

**Conclusions:**

This study shows that the ToC is a rewarding approach to framing dialogue with stakeholders and serves as a framework for planning, evaluation, learning, and reflection. We hypothesized that any future health IT implementation in primary care could adapt our ToC approach to their contexts with necessary modifications based on inherent characteristics.

## Introduction

### Background

Globally, digital health has been a tool of transformation for the delivery of health care services [1]. There is a plethora of health records in paper format resulting from the handling of clinical documentation across health care facilities in low- and middle-income countries (LMICs). In LMICs, few electronic health records (EHRs) exist at public primary health centers (PHCs), the first entry point for citizens or patients seeking essential health care services [2-4]. An EHR is defined as *“*a repository of information regarding the health status of a subject of care, in computer processable form*”* [5]. An EHR system can solve the bottleneck of paper documentation in health service delivery if it is successfully implemented, but poor implementation can lead to a waste of resources [6]. The study of EHR system implementation in LMICs is of particular interest to health stakeholders such as policy makers, funding agencies, and care providers because of the efficiencies and evidence base that could result from the appropriate evaluation of such systems [7]. Some progress has already been made regarding EHR implementation in LMICs, but sustainability and widespread adoption remain elusive [8,9]. A few examples of such developments in health care improvements include efficiency gains (such as quicker and more accurate reporting, reduced duplication of documentation, and quicker access to patients’ records), better patient tracking (such as immunization records and clinic attendance), and mobile health apps (ubiquitous access to remote care for patients) [10], an example of which is Virtual Doctors, a UK-based charity that specializes in telemedicine and provides remote medical advice to local health workers to reduce unnecessary hospital referrals. Currently, the charity is working with PHCs in Zambia and Malawi, where volunteer physicians, mostly from the United Kingdom, provide medical support through a mobile app. These volunteer physicians provide medically qualified advice where the local community only has a community health worker, leading to faster diagnosis and treatment [11]. Another example is iSanté, Haiti’s national electronic medical record system. This EHR system was implemented in 100 sites across Haiti primarily to support the delivery of the national HIV program [12]; it also supports antenatal care (ANC), delivery, and essential primary care services.

### Maternal and Child Health Care in Nigerian Primary Health Care

A significant health need in Nigerian primary care, in common with primary care in other LMICs, is maternal and child health care (MCH) [7]. Women and their children would usually attend the health facility for ANC, delivery, immunization, and family planning services [13]. It is essential to manage the health records of these patients or citizens effectively and efficiently to ensure effective clinical workflow and patient safety. Although paper-based health records seem to be structured in supporting care delivery, EHRs prove to be more consistent, readily available, and scalable for continuity of care [14].

### EHR Implementation in Nigeria

In high-income countries (HICs), there has been widespread adoption of EHRs, but this is not the case in many LMICs [1,9]. Despite the proliferation of mobile phones, the Nigerian health sector has not leveraged the advances in mobile technology for MCH delivery, unlike the health sectors in some other LMICs [15]. Similarly, the dominance of mobile apps in the financial and transportation sectors has not translated into the uptake of mobile health apps or telemedicine in the health sector [16]. So far, only a few hospitals in Nigeria have implemented an EHR system in some form [2,7]. However, there is a substantial use of EHRs for programs specific to diseases such as tuberculosis and HIV [17-21].

The challenges of health IT implementation in LMICs, especially Nigeria, include inadequate infrastructure, limited human capacity, brain drain, lack of enforcement of legislation and policies (political will), insufficient financial investment or incentives, and corruption-riddled systems [9,22-24]. Despite funding from the World Health Organization (WHO) and other funding agencies, the implementation is fraught with corruption. Private individuals and organizations in the health system divert the funds earmarked for these IT projects [25]. As a result of these acts, the patients or citizens who are beneficiaries do not get the intended quality of care and health outcomes [24]. Hence, funding agencies should include in funding applications a rider concerning how implementers monitor and evaluate the actual use and effect of resources provided. A very effective tool to achieve this is the development of a theory of change (ToC).

### ToC Fundamentals

The origins of the ToC can be traced to Chen and Rossi [26] and Weiss [27], who carried out extensive work in the area of theory-driven and theory-based evaluation. In particular, Weiss [27] popularized the term and modestly defined a “theory of change” as a theory of how and why an initiative will work. This definition seems simplistic; yet, it is foundational. ToC has evolved over the years, considering the ever-changing complexities in international development programs. In this study, we adopt the definition of ToC by the United Kingdom’s Department for International Development as *“*an outcomes-based approach which applies critical thinking to the design, implementation and evaluation of initiatives and programs intended to support change in their contexts*”* [28]. This definition relates to this feasibility study because this study aimed to bring about change by introducing EHR implementation in a primary health care context.

The ToC is both a process and a product [28-31]. The ToC process articulates the mechanisms of change. The process involves stakeholders who set a long-term goal and go in a reverse direction to specify assumptions and identify preconditions to achieve the desired outcomes [29]. This process leads to the product (ToC map) and is usually developed in versions before, during, and after program implementation. Although there is no single way to design ToCs, it can be asserted that good-quality ToCs should entail certain components such as long-term goals, assumptions, interventions, measurable outcomes, inputs, and outputs [32]. For a ToC to be deemed effective for any program or study such as this EHR implementation, it should fulfill these 3 criteria: it should be *credible*, *doable,* and *testable* [33]*.* The combination of assumptions from practitioners’ experiences, evidence from literature, findings from previous implementations, and program designer’s implicit logic substantiate the credibility of a ToC. In particular, articulating explicit assumptions about the feasibility of the EHR implementation helps to expose, test, and correct the program design logic. The assumptions are like theories that guide each ToC component and their interrelationships, and there is no one-size-fits-all set of assumptions. The assumptions vary from context to context and intervention to intervention [27,34]. On the basis of the specified assumptions, the activities carried out around the intervention will result in outputs, leading to indicators that can be measured to gain the confidence of relevant stakeholders: government, funders and nonprofits, health care workers, and ultimately patients [32].

### ToC and Other Relevant Frameworks

There are numerous frameworks used in health informatics, such as the logical framework (logframe) [35], DeLone and McLean (D&M) information systems (IS) success model [36], and examples presented in the WHO digital health monitoring and evaluation guide [37]. These frameworks have a broad purpose of assessing the maturity of an intervention over time but focus on specific criteria or dimensions; for instance, logframes involve logical designing, monitoring, and evaluating inputs, activities, outputs, outcomes, and impacts to achieve the desired results [38]. The logframe approach is very similar to the ToC approach in several ways. Logframes are useful and more linear [30]. Because of the complexity of the EHR intervention and the Nigerian environment, we found that ToCs were more adaptable with regard to capturing the ensuing complex interactions. The D&M IS success model measures the “complex-dependent variable” in IS studies [36]. This model is widely used to assess the interrelationship between critical evaluation dimensions of IT interventions, including information quality, system quality, service quality, system use or use intentions, user satisfaction, and net system benefits [5,39]. In the context of LMICs, the D&M IS success model has been validated by studying electronic hospital IS at 5 Nigerian teaching hospitals [39]. The WHO digital health guide is not a single framework; it examines several evaluation frameworks and illustrates how they could be practically used to support the implementation of digital health interventions in various contexts [37]. Having considered better-known evaluation frameworks, it is worth noting that the ToC scope goes beyond evaluation and covers planning, co-design, stakeholder engagement, and the linkage of causal pathways to individual outcomes. We used the ToC in this study to understand the problem as well as design and evaluate the intervention. The ToC applies to the whole life cycle of the intervention from the creation right through to the evaluation.

### Objectives

This study aimed to develop a ToC for the implementation of EHRs for MCH delivery in LMICs. The ToC approach will guide the entire transformation process from paper documentation to EHRs in the study context.

## Methods

### Setting

The study was conducted at the Festac PHC in Lagos, Nigeria, which has the highest number of physicians (7) and a wider range of health personnel than any other public PHC in Lagos State [40]. With the number of health care staff, the services provided, and operation hours (24 hours, 7 days a week), Festac PHC is a flagship public primary care center in Lagos State known for its role in reducing maternal and child health mortalities [41]. At this facility, patient information was written on paper and maintained in folders and health registers, which posed the issues of confidentiality, missing records, and inefficiencies. As of August 2019, Festac PHC employed 36 health care professionals (HCPs), who served an estimated population of 27,273 residents. There were additional HCPs at the other 16 private clinics and hospitals that serve the same population [40]. A research team funded by the Global Challenges Research Fund [42] through the University of Portsmouth worked with Festac PHC management to conduct a feasibility study for EHR implementation at the health facility. The health facility comprised 6 service departments, including the mother and child center, health records, consultation, general outpatient, laboratory, and pharmacy. At the mother and child center, midwives deliver MCH services and keep patient records in registers meant for services such as ANC, immunization, delivery, and family planning. At the health records unit, health information officers collect and maintain patient information with the help of registers, folders, and filing cabinets. The consultation unit consists of physicians (medical officers) who diagnose patients and keep patients’ clinical notes. In the general outpatient department, community health workers (nurses) observe and record patients’ vital signs. In the laboratory unit, a laboratory scientist and technicians run tests and maintain test data (specimen source, request, and results) of patients, aiding physicians and midwives in making diagnostic decisions. The pharmacy department consists of a lead pharmacist and pharmacy technicians who order, maintain, and dispense medicines. For this study, 14 participants (n=3, 21% physicians; n=5, 36% midwives and nurses; and n=6, 43% health records officers) were selected using purposive sampling because they were directly involved with patient data at Festac PHC [43]. The study commenced by conducting a remote scoping study in April 2019, which included readiness assessment (through an open-ended interview with the Festac PHC contact person), initial workflow analysis, and risk analysis through email or Skype consultation with the management team of Festac PHC.

### Design

**Figure 1:**
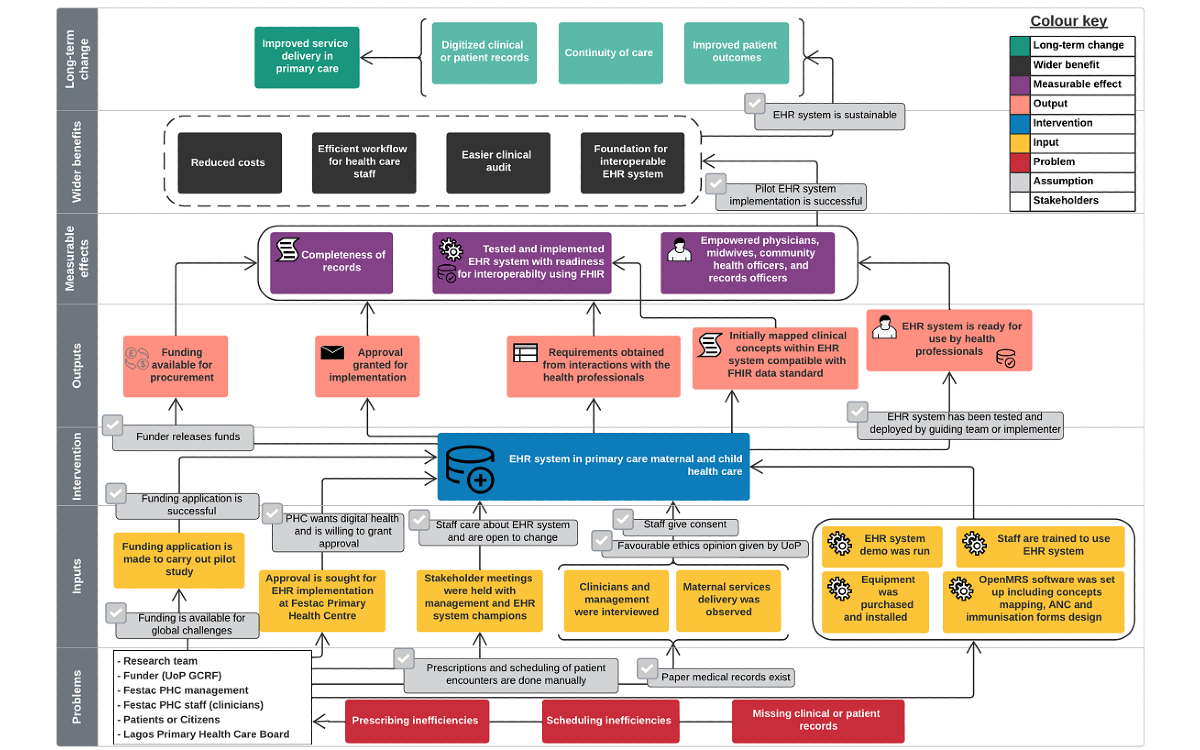
An initial version of the theory of change for the scheduled electronic health record (EHR) implementation at Festac Primary Health Centre (PHC). ANC: antenatal care; FHIR: Fast Healthcare Interoperability Resources; GCRF: Global Challenges Research Fund; OpenMRS: Open Medical Records System; UoP: University of Portsmouth.

**Figure 2:**
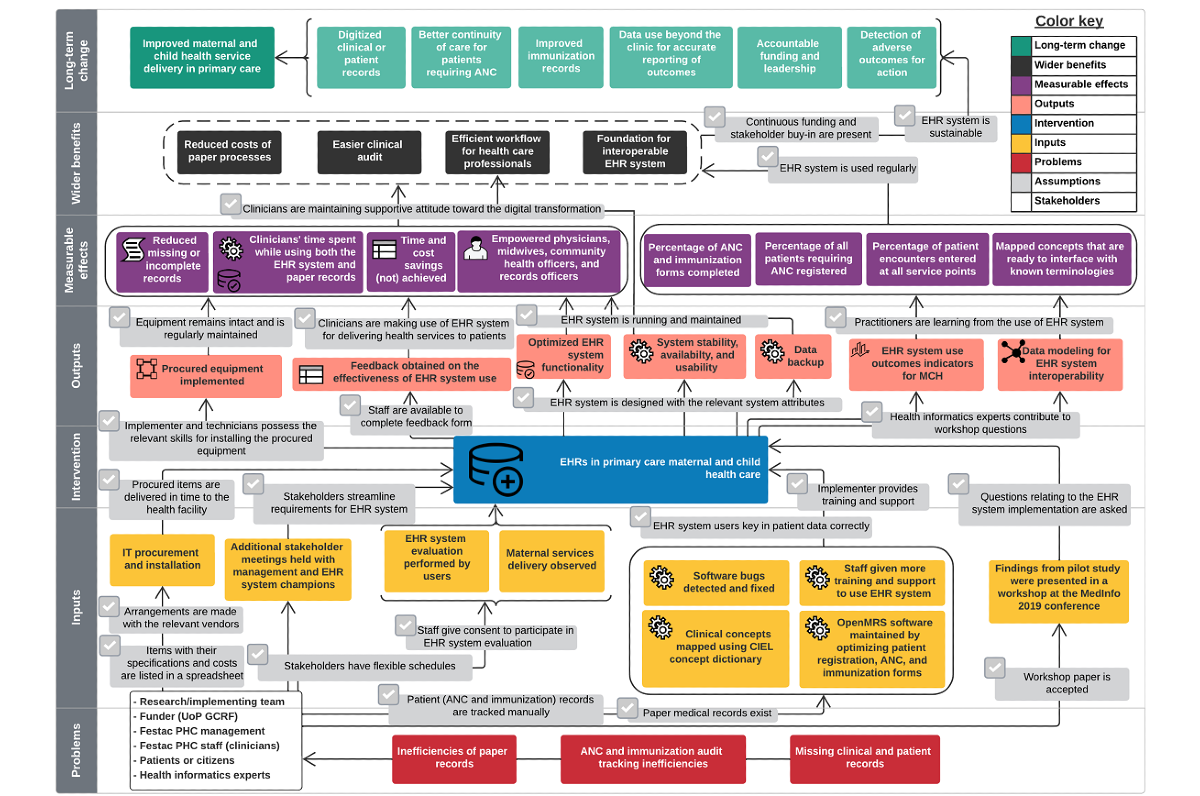
A revised version of the theory of change for electronic health record (EHR) implementation at Festac Primary Health Centre (PHC), including findings from a workshop at the MedInfo 2019 conference. ANC: antenatal care; CIEL: Columbia International eHealth Laboratory; GCRF: Global Challenges Research Fund; MCH: maternal and child health care; OpenMRS: Open Medical Records System; UoP: University of Portsmouth.

We used the ToC approach throughout the life cycle of the implementation to guide the pilot study and identify the preconditions needed to realize the long-term goal of the study [28,30]. Modifications were made from the initial version of the ToC to the revised version to reflect the realities of the implementation process. Because of the complex nature of EHR implementation, we developed and revised ToC maps with the relevant components. The research team developed the first ToC map (Figure 1) as an actual ToC based on evidence from literature, consultation with the local health information manager, and findings from previous EHR implementations. The ToC map illustrated the *problems* we were trying to solve, the *key* *stakeholders*, *assumptions*, *inputs*, *intervention*, *outputs*, *measurable effects*, and *wider benefits* of the implementation to realize the *long-term change* [44]. We developed a revised ToC map (Figure 2) to accommodate changes during and after the EHR implementation. These changes related to most of the ToC components and are documented under each component subheading in the Results section. We recognize that implementers should pay attention to sociotechnical issues, especially the interplay between patients’ realities and HCPs’ mental models and how these influence the EHR design and are represented within the system [45,46].

In the context of this study, the ToC components use these definitions:

- *Long-term change*: the desired goal the stakeholders want to achieve
- *Problems*: the challenges facing the current paper-based health records workflow as highlighted by the stakeholders
- *Stakeholders*: the people directly or indirectly involved or affected by the success or failure of the EHR implementation
- *Assumptions*: the beliefs that specify the underlying reasons for the logical connections that exist among the ToC elements. These beliefs are usually informed by research evidence, clinical practice, and the environment in which the change is taking place.
- *Inputs*: the activities or tasks carried out around the intervention
- *Interventions*: the initiatives or programs embarked on to influence the desired outcomes
- *Outputs*: the tangibles resulting from the inputs and the intervention
- *Measurable effects*: the immediate indicators that can be traced to the implementation process and are readily usable for evaluation. These measures can be quantitative or qualitative.
- *Wider benefits*: generalizable pointers that can guide the stakeholders with regard to the chances of implementing long-term change

The ToC approach is not immune to problems when used as an evaluation tool. Problems of theorizing, measurement, testing, and interpretation are not unusual [27]. To ensure rigor and evaluate the maturity of the implementation, we adapted the success criteria used in the studies by Deriel et al [12] and Fritz et al [47] to supplement the ToC approach. Textbox 1 outlines the categories considered for the success criteria of the implementation and provides definitions for each category.

We engaged the health practitioners and decision-makers at Festac PHC in designing, implementing, and evaluating the EHR system. In particular, the health practitioners at Festac PHC joined in developing the ToC versions, especially providing practical experiences that shaped the theories underpinning the ToC versions. This approach facilitated realistic interactions with the stakeholders and gave a proper understanding of the local context in which the study was conducted [48,49]. We had stakeholder meetings involving the heads of department and EHR champions at the PHC at the start and during the implementation process. Each stakeholder discussed the issues of the existing paper-based health record system and their expectations and experiences of the new EHR system, which validated the findings of the first ToC map. Subsequently, health informatics experts validated the revised ToC findings at the MedInfo 2019 conference in Lyon, France.

**Figure 3:**
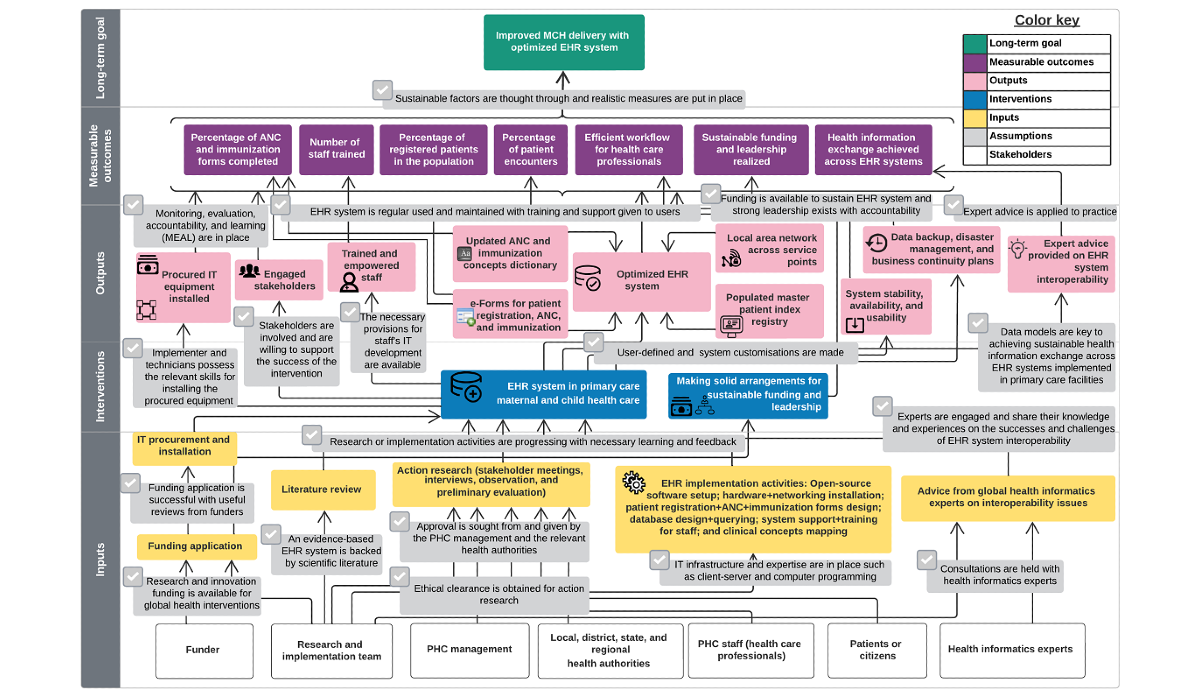
A generic version of the theory of change for electronic health record (EHR) implementation, without context-specific details. ANC: antenatal care; MCH: maternal and child health care; PHC: primary health center.

We developed a generic version of the ToC map (Figure 3) to reflect a holistic framework as a toolkit for relevant stakeholders who want to embark on this kind of intervention in similar contexts beyond Lagos, Nigeria. The stakeholders can adapt it for EHR implementations in primary care settings but need to pay close attention to inherent characteristics in these environments. Despite the nuances in different contexts, the process and steps involved in the creation of the ToC map are not to be ignored. Chen and Rossi [26] stressed the importance of giving adequate attention to understanding the implementation process and not being too concerned about whether the initiative has yielded excellent results.

**Categories for success criteria and their definitions for electronic health record implementation (adapted from Deriel et al [12] and Fritz et al [47]).**

**Categories and definitions**

- Ethics
  - Regulatory and cultural issues such as health data security, privacy, and confidentiality
- Political
  - Health policies and countrywide circumstances, including health care infrastructure, characteristics, ministries of health, and primary health care boards
- Organizational
  - Managerial circumstances within the organization itself, including human resources, skilled staff, or local buy-in; leadership and governance; project management and commitment to implementation; and data use
- Financial
  - Resources (including human and equipment) and funding
- Functionality
  - System features and functions, including modules, data handling, forms, and reports
- Technical
  - Infrastructure, software architecture, user interfaces, data standards, and privacy or security
- Training
  - Skills training as well as computer literacy and educational background and user support
- Sustainability
  - Transition from external stakeholder to local management across all categories, including financing

### EHR System Selection

Open Medical Records System (OpenMRS) is an EHR software program built for low-resource settings to improve health care delivery with the help of a global community that supports the software [50]. We selected OpenMRS as the EHR application for the pilot implementation because it is an open-source program and therefore freely available, which fits into the funding realities of LMICs, including Nigeria. The OpenMRS software source code can be modified and tailored to the needs of the particular context in which it is being used. It is an enterprise platform with flexible modules that have matured over time and been implemented in similar settings with a vibrant web-based community of developers and implementers [51,52]. We adapted existing OpenMRS modules to facilitate the identified use cases such as patient registration, outpatient clinic, laboratory, and mother and child clinic to manage clinical workflows. Moreover, we adapted UgandaEMR’s ANC and immunization e-forms to save development time and initial user-testing requirements.

### Ethics Approval

This study obtained a favorable opinion from the University of Portsmouth Faculty of Technology ethics committee (TECH2019-T.A-01). Participation in the study was voluntary, and participants were free to withdraw at any time without giving any reason. The participants provided written consent by completing a participant consent form. The study considered the security, privacy, and confidentiality of patient records from the outset. The paper health records were kept locked in a card room at the PHC. Although the reception area is positioned close to the card room, at busy times anyone could access the room with malicious intentions to cart away or damage the paper records. Hence, the EHR implementation took into account secure access to the electronic records by creating user accounts for relevant clinicians, ensuring that only users authorized by the heads of department could access the system [4].

## Results

### Overview

**Figure 4:**
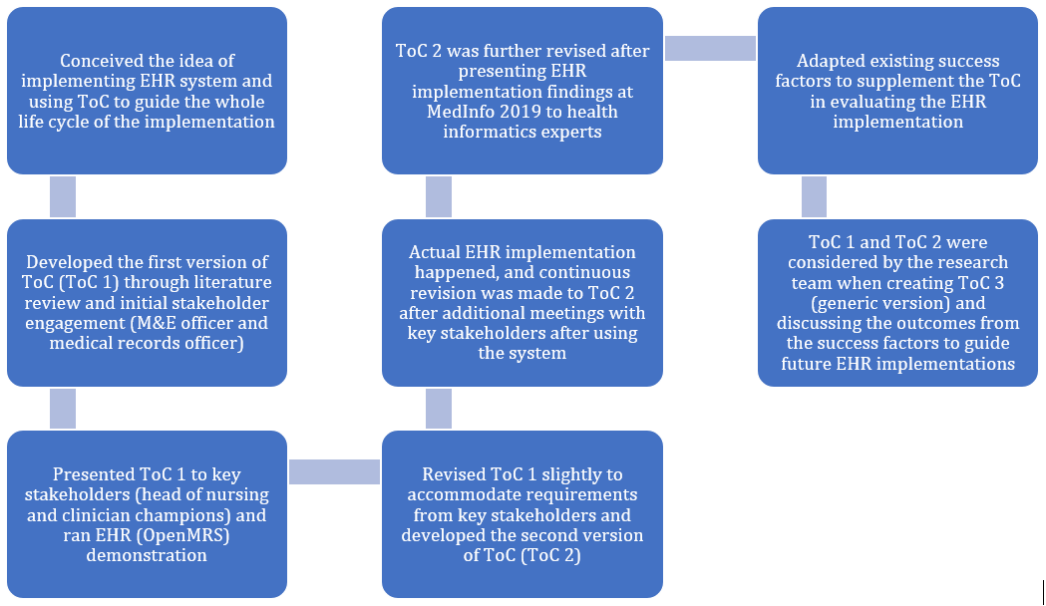
Complete theory of change (ToC) life cycle for electronic health record (EHR) implementation at Festac Primary Health Centre. M&E: monitoring and evaluation; OpenMRS: Open Medical Records System.

In this section, we report the complete ToC life cycle (Figure 4) for this study commencing from idea conception to the development of the initial ToC map and revised ToC map, illustrating how we accomplished the EHR implementation tasks at Festac PHC. At the same time, we hypothesize that program designers and relevant stakeholders can adapt the generic ToC map for EHR implementations in similar contexts. Subsequently, we provide a detailed narrative of the long-term change and identified preconditions from the ToC process. From this process, we produced a summary of the key successes and lessons learned alongside the study’s implications to evaluate the process (Multimedia Appendix 1).

### ToC Life Cycle

Figure 4 illustrates the entire ToC process for the EHR implementation and the key changes that occurred along the way. The ToC process is important because it helps to identify all the key stakeholders; for example, it helped to identify the significance of having clinical stakeholders evaluate the initial ToC. The conversations with the key stakeholders influenced the revised version of the ToC. Moreover, the process helped to identify problems early as well as the changes in direction for the EHR implementation, saving time, and cost.

### Long-term Change

Initially, the desired goal of the study was to achieve improved service delivery in primary care with the use of an EHR system. The allied goals were digitization of patient records, continuity of care, and improved patient outcomes. However, the overarching goal was slightly modified to accommodate practitioners’ assumptions. Hence, the long-term change is to achieve improved MCH service delivery in primary care using the EHR system. At Festac PHC, along with this long-term goal, the goals are digitization of patient records, better continuity of care for women using the ANC service, and improved handling of children’s immunization records. We theorize, with the generic ToC map, that the long-term goal is to improve MCH service delivery with an optimized EHR system based on the assumption that sustainability factors have been thought through, and measures have been put in place to achieve this goal.

### Assumptions

The initial ToC map served as the basis for the actual implementation, with preliminary assumptions emanating from the prior knowledge of the research team, the literature of existing EHR implementations, and initial conversations with the monitoring and evaluation officer and a medical records officer. The initial assumptions included the following:

1. Paper medical records exist.
2. Prescriptions and scheduling of patient encounters are carried out manually.
3. Funding is available for global challenges.
4. Funding application is successful.
5. PHC wants a digital health program and is willing to grant approval.
6. Favorable ethics opinion is given by the University of Portsmouth.
7. Funder releases funds for procurement of equipment.
8. Stakeholders care about EHRs and are open to change.
9. Practitioners give consent to be interviewed and observed at the health facility.
10. EHR system is tested and deployed by the guiding team and implementer.
11. Pilot EHR implementation is successful.
12. EHR system is sustainable.

After the actual EHR implementation, the initial ToC map was revised to reflect the real changes encountered during the pilot study; for example, priorities for the EHR system shifted from scheduling and prescription to ANC and immunization. At the time of developing the initial ToC map, the Festac PHC stakeholders had identified the need for booking patient appointments and producing prescriptions electronically with the EHR system. However, after the face-to-face stakeholder meeting at the health facility, the practitioners noted that e-forms for ANC and immunization were their immediate needs for the EHR system. Another change to the ToC revision was the shift in networking design from the cloud to a local area network. This shift was due to connectivity problems and a lack of guarantees from the management regarding sustaining the internet subscription payment. This is the dominant approach to EHRs in LMICs because few of the smaller sites can guarantee reliable internet connectivity for cloud-based use, although certain LMICs do this well [14,53].

In addition, the revised ToC included findings from the research workshop (MedInfo 2019 conference), where the EHR use outcomes from the pilot study were presented. Global health informatics experts offered advice at the workshop, during which it was emphasized that data models are key to realizing effective communication exchange across digital health systems by adopting the appropriate interoperability standards for MCH, well-known examples of which are Fast Healthcare Interoperability Resources [54] and OpenEHR [55]. In addition, the drivers for an interoperable EHR system differ between LMICs and HICs; for example, LMICs focus mainly on aggregate data from the health information system for disease control, population health monitoring, and health policy and planning. Funders use these aggregate data to drive health financing and, in some cases, to fund EHR implementations. However, HICs pay more attention to the quality of care, continuity of care, and precision medicine. In addition, adequate infrastructure and accountable funding were identified to be key preconditions needed for a sustainable EHR implementation. In sum, toolkits are important in shaping EHR implementations for MCH services.

Although some *assumptions* stay the same, others were modified. Textbox 2 illustrates these assumptions and how they were generated.

For the improvement of MCH services to be achieved, it was assumed that the EHR system was sustainable. The EHR system needs to be used regularly to bring about the broader benefits of its implementation.

**Assumptions and their sources.**

**Assumptions and sources**

- Antenatal care and immunization records are tracked manually
  - Practitioners
- Items with their specifications and costs are listed in a spreadsheet
  - Electronic health record implementation
- Arrangements are made with the relevant vendors
  - Program designer
- Procured items are delivered in time to the health facility
  - Electronic health record implementation
- Implementer and technicians possess the relevant skills for installing procured equipment
  - Policy makers and program designer
- Stakeholders have flexible schedules
  - Practitioners
- Staff give consent to participate in the electronic health record system evaluation
  - Practitioners
- Stakeholders streamline requirements for electronic health record system
  - Practitioners and program designer
- Implementer provides training and support
  - Policy makers, practitioners, and program designer
- Electronic health record users key in patient data correctly
  - Practitioners and program designer
- Workshop paper is accepted
  - Program designer
- Questions relating to the electronic health record implementation are asked by workshop participants
  - Health informatics experts and program designer
- Health informatics experts contribute to workshop questions
  - Health informatics experts and program designer
- Staff are available to complete a feedback form
  - Practitioners
- Electronic health record system is designed with the relevant system attributes
  - Practitioners and program designer
- Hardware equipment and electronic health record system software remain intact and are maintained regularly
  - Policy makers, practitioners, and program designer
- Clinicians are making use of electronic health records regularly for delivering health services to patients
  - Practitioners and policy makers
- Stakeholders are learning from electronic health record use and data
  - Policy makers, practitioners, and program designer
- Clinicians are maintaining a supportive attitude toward digital transformation
  - Policy makers, practitioners, and program designer

### Wider Benefits

On the basis of the assumption that the pilot EHR implementation is successful, there would be benefits accrued to Festac PHC. These benefits include reduced costs of paper processes, including expenses for stationery; efficient workflow for the health care staff; easier clinical audit of patient records; and readiness for a sustainable EHR system. The sustainability of the EHR system will enable effective health information exchange as the use of EHRs becomes widespread over time.

### Measurable Effects

**Figure 5:**
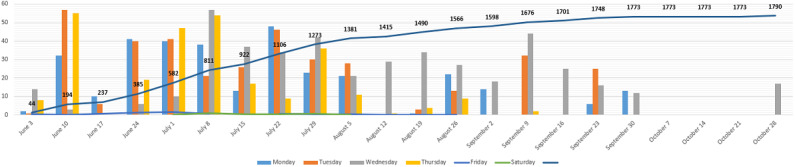
Cumulative daily and weekly data entry with regard to patients registered on the electronic health record system.

It was anticipated that the availability of the EHR system alongside the surrounding outputs would result in the completeness of health records, which could be measured against the use of paper health records by the health practitioners. Another potentially measurable effect concerns clinician time spent using both paper and EHR systems [56]. During the implementation, we found that clinicians spent more time using both paper and electronic systems simultaneously, which affected the EHR system’s complete records outcome. We enrolled 14 clinicians to use the EHR system, and Figure 5 shows the rate of EHR system adoption and use for the study’s first phase lasting for 5 months (June 2019 to October 2019) from the time the system went live. A total of 2799 encounter forms were completed on the EHR system; 1790 (63.95%) patients were registered, with an equivalent number of patient registration forms being completed. ANC and immunization encounter forms (198/2799, 7.07% and 309/2799, 11.04%, respectively) were completed. Vital signs (325/2799, 11.61%) and visit notes (177/2799, 6.32%) were entered into the EHR system. Of the 325 vital signs forms, 148 (45.5%) consultations were not recorded using the visit notes because some physicians only used paper notes. A major system downtime occurred from October 3 to 29, 2019, which affected data entry. Longer-term success factors, which are yet to be measured, are the realization of funding sustainability and accountable leadership, as well as health information exchange achieved between the EHR system and other health IS.

### Outputs

The study received a letter of approval for the implementation from the local authority. This approval enabled the release of funds and the travel of a research team member (TA) to the health facility. The funder released the funds to procure the IT equipment needed for the study. The interactions with the health practitioners made it possible to obtain the requirements to design and develop the intervention. After we incorporated the active inputs of various stakeholders, the EHR system was ready for use by the health practitioners. The outputs in the revised ToC map were procured equipment, feedback from EHR use, optimized EHR system functionality, EHR use outcomes indicators for MCH, and data modeling for EHR system interoperability. Other key outputs were the critical system attributes (such as system stability, availability, and usability) and full and incremental data backup of patient records to the cloud. In the event of system damage, fire, flooding, or any adverse incidents, the PHC can restore the records from the backup.

### Intervention

The main intervention for this study was the introduction of an EHR system in primary care MCH services. Initially, problems were perceived based on explicit and implicit assumptions about paper medical records and prescriptions and scheduling of patient encounters being carried out manually. After face-to-face stakeholder meetings on site, the practitioners were of the unanimous opinion that prescribing and scheduling inefficiencies were not the priority issues; rather, priority should be accorded to paper records handling, ANC and immunization-tracking inefficiencies, and missing patient records. These problems validated the introduction of the EHR intervention at Festac PHC.

### Stakeholders

The stakeholders are the research team (TA, PS, and HF), funder, Festac PHC management (local authority), primary health care board, health care practitioners, patients, and health informatics experts. They carried out several activities at various stages of the study. The research team made some informal contacts with the local primary care facility authorities to understand their problems and the desired long-term outcomes. The team reviewed existing studies to gain background knowledge of previous EHR implementations in similar contexts. After the review, the team developed the initial version of the ToC map, informed by the explicit assumptions of the practitioners and implicit assumptions gleaned from previous implementations. The research team prepared a funding application and sought approval for the pilot study. Both the funder and the local health authority approved the pilot study.

### Technical Implementation

There was a demonstration of the OpenMRS software during the first stakeholder meeting. The activity helped the practitioners to have a feel of how the intervention works. Before this meeting, the contact person from the PHC had been testing the demonstration version of the EHR system; they gave feedback on what the PHC specifically wanted. The main technical components of OpenMRS are the database (eg, data concepts mapping, backups, and security) and the EHR software (clinical modules and customizations). The research team initially designed a cloud solution before the implementation but changed to a local area network design because of poor internet access at the health facility. Through a combination of on-site and remote support, the research team contributed to installing and configuring the software. The equipment included laptops, a desktop PC (dedicated server), networking equipment (16-port Ethernet switch, wireless router, Category 6 cables, and RJ45 connectors), a power inverter (to provide power for the server when electricity from the national grid and generator set is unavailable), and a printer.

## Discussion

### Principal Findings

This study shows the value of the ToC process for robust planning, analysis, and evaluation of EHR implementation complexities, as well as challenging the assumptions of all stakeholders. The process requires logical reasoning, effectively engaging stakeholders in drawing implicit assumptions, designing the preconditions, and mapping the ToC backward from the long-term goal to inputs. Political factors play a role in influencing what practitioners say about their beliefs or theories regarding the desired change. The practitioners may have concerns about the management’s disapproval of their assumptions [33]; for example, we asked the HCPs about the leadership style of their line managers and the effect it has on their use of the EHR system. Some (7/14, 50%) of the HCPs made positive comments about their managers. Although it is possible to have all-positive feedback about leadership styles in a typical work setting, the lack of concerns or negative comments may suggest desirability bias or groupthink [57].

A ToC is useful in articulating assumptions made about a program or intervention to achieve its desired results. We generated assumptions from peer-reviewed evidence (documents and prior research); experience and views of practitioners and other stakeholders such as funder, government, and policy makers; and logical reasoning (Textbox 2). However, it can be problematic to test assumptions even when they are explicitly stated. Problems such as measurement, generalization, and validation usually plague program theory [27]. Our study extensively evaluated the ToC-based implementation using previously defined success criteria across multiple dimensions of implementation and use (Multimedia Appendix 1) [12,47], which is a methodological innovation in LMIC settings because of the wide range of evaluation criteria. However, combinations of some individual criteria have been used. Certain authors have argued that theory-based evaluation such as the ToC is more a methodology than a theory because it uses different research methods (eg, randomized controlled trials, interviews, and workshops) for its development [30,44]. Weiss [33] argues that the ToC is an approach and a theory because it is built on assumptions (beliefs), preconditions, inputs, and outputs, which influence the way people behave.

Again, the ToC approach is particularly useful in capturing the complexities of a program relating to its outcomes, outputs, inputs, and activities to bring about long-term change by using relevant interventions [58]. The research team engaged the relevant stakeholders by asking them to share their experiences and practices (explicit assumptions). We drew out the implicit assumptions, which were not obvious to the practitioners and experts, through interviews and a workshop (findings to be published), and then modeled these assumptions and combined them with evidence and logic, all of which were put together in readiness to transfer into practice.

### Reflections Based on Experiences of EHR Implementations in Other LMICs

Despite Festac PHC being an early adopter of the EHR system and the only one among public PHCs in Lagos State, the management has not done enough in terms of funding the infrastructure and ensuring its sustainability. The issue of funding and other EHR implementation challenges are not peculiar to the Nigerian context; rather, they are applicable to different LMIC contexts [51,53]. Comparison evaluations of EHR systems in LMICs were provided by 2 papers, published in 2017 and 2018 (Multimedia Appendix 2 [51,53]). Although there is anecdotal evidence of EHR implementations across Nigeria, there is no known peer-reviewed evidence of OpenMRS implementation in the country. As of June 2021, the OpenMRS HIV Reference Implementation initiative funded by the Centers for Disease Control and Prevention is supporting >1000 site rollouts of OpenMRS in Nigeria as well as improvements in the user interface, reporting, and other initiatives [59]. A recent paper [60] tried to examine the impact of OpenMRS implementations globally over a 15-year period, but no concrete evidence on Nigeria was available, except for some brief mentions. This study should help to address this gap, especially where public primary care in Nigeria is concerned.

Multimedia Appendix 2 compares findings from OpenMRS implementations in 3 LMICs (Nigeria, Sierra Leone, and Kenya), inclusive of this study (Festac PHC in Nigeria). Common findings across the 3 studies related to data collection, staff training, and infrastructure. These studies showed that EHR use results in clinical workflow efficiencies. At the same time, the studies discussed the challenges encountered during implementation, which centered mainly on inadequate infrastructure, funding, dedicated IT support, and stakeholder buy-in. A significant issue across the 3 EHR implementations is sustainability, and our Nigerian (Festac PHC) study used the ToC approach to underscore this issue extensively. Despite their successful completion, the implementations did not continue beyond the first or second phase. Hence, stakeholders must pay close attention to sustainability issues before embarking on EHR implementations in LMICs.

### Reflections Based on Experiences of EHR Implementations in HICs

Policy makers and politicians in LMICs can learn from countries that incentivized EHR adoption by providing implementation funds to health facilities. A prime example is the United Kingdom, where the EHR adoption rate in primary care, particularly general practitioner (GP) practices, is nearly 100% [61,62]. Among other factors, financial incentives from the government have proven to be an effective impetus for EHR implementation across GP practices. For many years, thought leaders in the GP profession have collaborated with the government to provide incentives for digitizing practices and eliminating barriers. Hence, GPs were more willing to use EHRs than hospital physicians, helping the former leverage the successful health IT intervention [62]. However, despite the successful EHR adoption rate by GP practices in the United Kingdom, the system has its shortcomings: it sometimes fails as patients show up at the community pharmacy expecting to pick up their medications only to find that the electronic prescription has not reflected in the pharmacy system. This issue can often delay treatment for patients, especially on weekends when GP practices are closed, and the pharmacy team chases prescriptions. The GP’s on-call team can usually access the system and fax the prescriptions to the pharmacy, but the effectiveness of this process varies across the United Kingdom.

The US government program based upon the Health Information Technology for Economic and Clinical Health Act of 2009 provides financial incentives to physician practices and hospitals to foster digital health implementation and improve the quality of care for patients. These incentives have since led to the widespread adoption and meaningful use of EHR systems across all levels of health care in the United States, with the resultant digital health transformation and improved clinical outcomes [63-65]. However, rapid implementation of existing EHR systems has been associated with many challenges in workflow, usability and physician stress or overload. The UK model of adoption of primary care EHR systems may be better in terms of a limited number of carefully vetted systems, low costs, and robust interoperability with many hospitals; for example, in West Yorkshire [66].

### Reflections on Data Entry at Festac PHC

Inconsistencies in EHR data entry during patient encounters occur because of several factors, including human, organizational, and system factors. The willingness of clinical staff to use the new system was lacking because of the perception that the system would add to their existing workload, reflecting the realities of data entry operations and the shortage of health workers in LMICs [67]. Only a few HCPs were keen on using the system. Hence, little or no data entry is completed if the active HCPs are not on duty. Sometimes, the HCPs attend staff verification exercises, leaving the EHR system in the hands of casual staff who do not have permission to use it because of clinical accountability requirements. Lack of leadership motivation or incentive to use the system could prevent health information officers, physicians, nurses, and midwives from understanding the need to work on data entry. System downtime happens occasionally; when this happens, there is no health IT support technician on the ground to resolve the issue, and hence the PHC relies on the implementer, who, although not contractually obliged, may sometimes help out. To resolve system issues, the PHC management could employ an IT support technician on a full-time or part-time basis, but the management should be keen and be ready to include the employment cost in the clinic’s budget. In a recent review on the importance of primary care records in LMICs, we found that there seems to be a particular challenge with EHR data collection in primary care organizations [68]; for example, MCH EHR data collection was challenging because of local factors such as the level of technology available for data entry at the point of childbirth. Hence, this is a larger problem for people who run modest primary care EHR systems in LMIC settings, a problem not specific to Nigeria. This implementation study successfully demonstrated improvements in MCH services data collection. However, the lack of effective human, organizational, and system support is responsible for inconsistent data entry in the EHR system, leading to poor clinical benefits and inaccurate reporting.

The ToC approach gave insights into the potential causes of the breakdown of the system, such as the issues concerning regular use and data entry by key staff, which allowed for provision of additional planning and training. A simple *cost-benefit* approach to framing the overall implementation process to determine the likely gains (value) to staff, patients, health systems, and funders would be helpful. It would be valuable to determine whether these costs outweigh the challenges of learning to use the system and the pain of working on data entry. In addition, the proposed investment in infrastructure and support could be balanced by the concrete benefits. The costs often fall on staff working on data entry who do not benefit much from the outputs. Hence, the combined effect of the utility of an application and ease of use gives stronger predictability for actual use, which is incorporated in the D&M model.

There is a growing interest in alternative data entry approaches, including the “scribe” model (in US primary care) [69], natural language processing–enabled data capture, and optical mark recognition (OMR). These alternative approaches could address the issue of clinicians’ avoidance of using the EHR system. The “scribe” model introduces a way of working where a human scribe (a volunteer or health professional) manually enters the applicable information such as observations, diagnosis, and test results into the EHR during the patient visit as spoken aloud by the physician or nurse [70]. However, this could affect clinical data quality because the scribe might not be a suitably qualified clinician and prone to making data entry errors, which, in turn, could affect health outcomes. Natural language processing data capture applications allow HCPs, especially physicians, to capture structured data with unstructured dictation into the EHR [71]. OMR is a nondictation, scanning method of data capture where the OMR software processes paper clinical forms that have been scanned with a modest office scanner or low-cost document camera [72]. This approach ensures that clinicians who record clinical data on paper do not also have to enter the data once or twice in other records. It requires stability of systems, a person to oversee the scanning and data extraction, and user confidence. It might develop as a model to overcome a data entry backlog in the EHR system, increasing the value for clinicians, particularly if recent improvements in optical character recognition software can be shown to be effective in interpreting structured handwriting.

### Limitations

This study includes several limitations with regard to developing the ToC. First, the research team was extensively involved in developing and revising the ToC map, which may have contributed to a social desirability bias. Second, the first author (TA) mainly worked on the analysis of the ToC maps under the guidance of the last author (PS) and the second author (HF). We would have engaged the HCPs and stakeholders in the analysis, but they were not well versed with the technicalities of the ToC approach. Future studies will ensure that HCPs are familiarized with the ToC analysis. The relevant stakeholders were fully engaged in the clinical, data collection (interviews and observations), and managerial aspects of the design.

### Conclusions

This research presented the ToC as a rewarding approach to framing dialogue with stakeholders. It functioned as a valuable framework for planning an EHR implementation and the steps needed to define the requirements and success factors, likelihood of longer-term success, and evaluation metrics. For new implementers, knowing how to structure this implementation process could be very useful. Future health IT implementation in primary care can adapt the ToC approach to their contexts with necessary modifications based on inherent characteristics. The pilot EHR implementation served as a small-scale foundation that can support health information exchange and as a digital health exemplar for other PHCs in Lagos State and Nigeria. Other health care providers can learn from, and build on, the implementation to support the delivery of MCH and other health services. Furthermore, the pilot EHR system represented a digital enabler that provides computable and machine-readable health data, the necessary first step toward more complex aspects such as interoperability, clinical decision support, and a learning health system. Further work is needed to extend the scope of the implementation to cover other public PHCs. There is a need to secure more funds for additional infrastructure alongside solid leadership to ensure sustainability and scalability. In addition, it will be helpful to explore the interoperability of health data across public PHCs by designing a national health data model for an MCH services data set. The model should be based on established data standards and an examination of the preconditions and drivers for implementing such a model and build on existing work on clinical decision support for MCH services [73].

## Appendix

Multimedia Appendix 1 Summary of successes achieved and lessons learned from the pilot study at Festac Primary Health Centre, as well as implications for electronic health record implementations in Nigeria and other low- and middle-income countries.

Multimedia Appendix 2 A comparison of electronic health record implementation findings from 3 studies conducted in low- and middle-income countries.

## Abbreviations

ANC: antenatal care
D&M: DeLone and McLean
EHR: electronic health record
GP: general practitioner
HCP: health care professional
HIC: high-income country
IS: information systems
LMIC: low- and middle-income country
MCH: maternal and child health care
OMR: optical mark recognition
OpenMRS: Open Medical Records System
PHC: primary health center
ToC: theory of change
WHO: World Health Organization
