# Supplementary material for: Implementing Electronic Health Records in Primary Care Using the Theory of Change: Nigerian Case Study
Source: JMIR Med Inform. 2022 Aug 11;10(8):e33491. doi: 10.2196/33491 (PMC9412900; doi:10.2196/33491)
Supplement: Multimedia Appendix 1 [file medinform_v10i8e33491_app1.docx]

Multimedia Appendix 1. Summary of successes achieved and lessons learned from the pilot study at Festac Primary Health Centre (PHC), as well as implications for electronic health record (EHR) implementations in Nigeria and other low- and middle-income countries (LMICs).

| Categories | Key case study successes | Lessons learned and implications |
| --- | --- | --- |
| Ethics | - The Nigeria Data Protection Regulation 2019 guidelines were enacted into law at the time of this study. This study complied fully with the General Data Protection Regulation 2016 guidelines applicable within the United Kingdom - Log-in credentials were created for all users across departments, and unauthorized access was prevented. This measure ensured the security of patient records in terms of privacy and confidentiality | - The concept of patients’ ownership of data is not widely appreciated |
| Political | - The EHR implementation fits into the vision of the National Health ICT^a^ framework for Nigeria, which states that “by 2020, Health ICT will help enable and deliver Universal Health Coverage” [22] - The local authority bought into the project and provided relevant support needed to proceed with the implementation without any interference from political figures | - We experienced some delay in receiving approval from the local authority for the EHR implementation because of the number of persons needed to sign the permission request. The primary contact from the PHC made efforts to get all the relevant parties to go through the paperwork. Building informal relationships and maintaining communication with reliable local contacts is vital, particularly those keen on the digital health transformation agenda. Sometimes, this approach works differently in LMICs and HICs^b^ |
| Organizational | - The management of the Festac PHC granted the necessary approval to the research team to commence the pilot study - The health professionals, especially the head of nursing and HIOs^c^, showed significant commitment to adopting the EHR system - Efficient workflow for health professionals | - Certain physicians and midwives were unwilling to use the EHR system initially until they were persuaded or incentivized by the management. The clinicians perceived that EHR use would add to their ever-busy workload, and staff shortage was an issue. Although the benefits of the EHR system were visible, it was hard for the heads of units to convince the clinicians to use the EHR system because of organizational politics - It was challenging to go fully electronic; hence, the HIOs maintained both paper and electronic records until they were confident that the patient records matched in both formats |
| Financial | - Secured funding for the feasibility study - Procured the IT equipment, including servers, laptops, and networking hardware - Reduction in cost of IT infrastructure that the Festac PHC would have incurred | - The initial cost of implementation was high, and funds mainly came from the funding partner. Supplementary funding from the local authority directing Festac PHC would have moved the project forward significantly; for example, suitable financing would have enabled the purchase of additional laptops for the consulting rooms, immunization points, laboratory, and pharmacy. Access to the EHR system with the required infrastructure by all service points during the patient visit will ensure completeness of records for proper reporting and improved delivery of patient care. Moreover, additional funds could have been used to pay for a continuous broadband subscription, which would have enabled remote technical support for the EHR system |
| Functionality | - Implementation of patient registration, outpatient, laboratory, and reports modules - ANC^d^ and immunization e-forms were designed efficiently to encourage data entry by HIOs - Easier clinical audit tracking for antenatal care and immunization of patients - The EHR system has helped Festac PHC to keep more complete records of patients or citizens | - A research team member (TA) adapted UgandaEMR’s ANC and immunization e-forms instead of reinventing the wheel, which seemed like an important way to reduce cost barriers in the software configuration and in implementation costs. There is little or no published evidence on OpenMRS^e^ implementation for primary care in Nigeria. The closest example to model for the implementation was the UgandaEMR customization of OpenMRS for Uganda—it was freely available on the web on a test server for implementers within the Ugandan contexts. The Festac PHC stakeholders were convinced and agreed to adapt the UgandaEMR forms. Although it served as a helpful template, modifications were made for the Nigerian context. Despite apparent similarities, there was little real overlap in the requirements and required forms; for example, the ANC concepts differ between Nigeria and Uganda; hence, the expertise of the M&E^f^ officer and the midwives was sought to understand the concepts, which helped in successfully coding them in the EHR system - JavaScript was used to optimize the e-forms (hide and show content) to encourage efficient data entry for HIOs and reduce the boredom of scrolling through lengthy webpages each time ANC and immunization records are entered. This optimization is a significant improvement on UgandaEMR’s static e-forms |
| Technical | - LAN^g^ design was implemented across service points at the PHC - The enterprise edition of OpenMRS was successfully installed using a WAR^h^ file | - Work on the initial cloud design was halted because of poor internet access (4G^i^) at the clinical site. Moreover, there was a lack of commitment to funding wireless subscriptions beyond the amount budgeted. Future implementations need to have an alternative network design plan (a wired LAN connection would be the most reliable) - There were compatibility issues with Java Runtime Environment, MYSQL server, and Apache Tomcat. These issues took approximately 3 days to resolve until the compatible versions were identified. Future implementers need to be aware of potential compatibility issues with software applications before implementation and, at the same time, prepare to be tenacious or dedicated because it can be a discouraging experience, especially when expectations are high for a timely implementation delivery |
| Training | - Clinical staff were trained to use digital health - Technical support was provided during and shortly after the implementation | - Health practitioners could not continue to support the EHR system because there were no technical staff members on the ground. The lack of technical support affected the smooth running of the system, and the clinicians were left at the mercy of the busy implementer. The PHC management should make provisions for periodic staff training and system support to ensure continuous staff learning and effective running of the EHR system after the implementation |
| Sustainability | - Implementation of a wired LAN architecture at the health facility so that there is local networking in place for the different client locations to access the server - Internet connectivity for 12 months to support data backup, synchronization, clinical research, reporting, and remote support for the EHR system - Installation of a 1 kVA power inverter with a 200 Ah battery to run the server for 10 hours in the absence of electricity from the national grid and generator - Purchase of laptops with a battery life of 6 hours each for operations such as data entry - Implementation of a routine backup of the database containing patient records - Equipment was handed over formally to the M&E officer (primary contact for the PHC) with a letter containing the equipment list and signatures of relevant stakeholders. This formality was completed to ensure that the equipment is maintained properly, although this is not guaranteed | - The practical application of the ToC^j^ approach provided a better understanding of the successes and failures experienced in this study. Although the implementation collapsed, the benefits of the implementation were sustained. The sustained improvements included trained health care professionals, a change in mindset from using paper systems toward digital health transformation, and using the project’s laptops to collect aggregate data for a DHIS2^k^-based national health information management system - The funding stopped, and the PHC management did not continue with the financing. Hence, there should be a firm commitment from the local authority to provide supplementary funding for the operation and maintenance of the EHR system, especially before the project initiation and after the implementation. The ToC process helped to reveal how accountable funding is a necessary precondition to the sustainability of a digital health implementation - The M&E officer was transferred to another PHC 6 months after the implementation. This staff movement created a knowledge gap in maintaining the system |

^a^ICT: information and communication technology.

^b^HIC: high-income country.

^c^HIO: health information officer.

^d^ANC: antenatal care.

^e^OpenMRS: Open Medical Records System.

^f^M&E: monitoring and evaluation.

^g^LAN: local area network.

^h^WAR: web application resource.

^i^4G: fourth-generation wireless.

^j^ToC: theory of change.

^k^DHIS2: District Health Information System 2.
