# Supplementary material for: Implementing Electronic Health Records in Primary Care Using the Theory of Change: Nigerian Case Study
Source: JMIR Med Inform. 2022 Aug 11;10(8):e33491. doi: 10.2196/33491 (PMC9412900; doi:10.2196/33491)
Supplement: Multimedia Appendix 2 [file medinform_v10i8e33491_app2.docx]

Multimedia Appendix 2. A comparison of electronic health record (EHR) implementation findings from 3 studies conducted in low- and middle-income countries.

|  | Nigeria (this study) | Sierra Leone [53] | Kenya [51] |
| --- | --- | --- | --- |
| Year of implementation | - 2019 | - 2015 | - 2014 |
| Location or site | - Festac PHC^a^, Festac Town, Lagos | - Ebola Treatment Centre, Kerry Town | - Public hospitals (county referral hospital and health centers in Machakos and Baringo counties) |
| Aim or objectives | - To develop a ToC^b^ to assess the feasibility of EHR implementation for MCH^c^ delivery in low- and middle-income countries | - To rapidly develop OpenMRS^d^-Ebola, an open-source Ebola EHR system that was implemented in 2015 at Save the Children’s Kerry Town Ebola Treatment Centre - To describe the experiences, lessons learned, and recommendations for design and implementation of EHR systems in future health emergencies | - To present a descriptive case study of the implementation of an open-source EHR system at public health care facilities in Kenya |
| Methodology | - Applied the ToC approach - Adapted some success factors to supplement the ToC approach in evaluating the maturity of the EHR implementation | - 4-component strategy: - Agile software methodology - Recruited team members with diverse skills and experiences - Iterative design based on usability, speed, and clinical needs - Regular communication and feedback between the operations and development teams | - Conducted the following exercises: - A landscape review of existing literature concerning eHealth policies and EHR development in Kenya - Informal discussions with the ministry of health, the World Health Organization, and implementing partners - A series of visits to implementing sites - Semi-structured individual interviews and group discussions with stakeholders to produce a historical case study of the implementation |
| Software | - OpenMRS | - OpenMRS-Ebola | - OpenMRS |
| Hardware | - Laptops, server, generators, and power inverter | - Solar-charged tablets, laptops, servers, and generators | - Laptops, 0 clients (all-in-one computer terminals), server, solar power equipment, and generators |
| Networking | - Wired network | - Wireless network | - Wired and wireless network |
| Scale (pilot or at scale) | - Pilot | - Emergency or crisis | - At scale |
| Project completion | - Phase 1 completed | - Phases 1 and 2 completed; phase 3 partially completed (not deployed) | - Phases 1 and 2 completed |
| Program (MCH and Ebola) | - MCH and essential primary care services (outpatient clinic, pharmacy, and laboratory) | - Ebola | - MCH and essential primary and secondary care services (outpatient, pharmacy, laboratory, inpatient, specialized clinics, and community health care system) |
| Level of care (primary or secondary) | - Primary | - Primary and secondary (treatment center) | - Secondary (phase 1) and primary (phase 2) because of scaling back |
| Key findings | • ToC: 3 ToC maps; of the 2799 encounter forms completed, there were 1790 (63.95%) patient registrations, 198 (7.07%) ANC^e^ and 309 (11.04%) immunization completed e-forms, and 325 (11.61%) vital signs and 177 (6.32%) visit notes entered into the EHR system  • Ethics considerations: log-in credentials were created for all 20 users across departments, and unauthorized access was prevented  • Political: local authority buy-in  • Organizational: management and staff buy-in  • Financial: procured the IT equipment, including 1 server, 10 laptops, and networking equipment  • Functionality: implemented patient registration, outpatient, laboratory, and reports modules; designed ANC and immunization e-forms  • Technical: local area network design was implemented across service points at the PHC  • Training: 14 clinical staff were trained to use the EHR system, technical support was provided  • Sustainability: internet connectivity for 12 months to support data backup; formal handover of equipment with letter of agreement for sustainability | • Training: 100 clinicians were trained; estimated cost: US $187,000  • System use:   - 112 patients were registered - 569 prescription orders were placed - 971 medication administrations were recorded   • Paper records:   - 15 errors during patient registration - 553 of 569 prescription orders correctly matched | • System (infrastructure): 15 laptops, 1 central server, 5 IT staff, 4 IT interns, and 1 software support company  • People: trained staff at 4 health facilities  • Process: data entry, accuracy, and integrity carried out by clerks  • Products: optimized software modules, including patient registration, outpatient, inpatient, laboratory, pharmacy, health records, and hospital inventory |
| Notable challenges | • Ethics: no concept of patients’ ownership of data  • Political: delay in receiving local authority approval  • Organizational: clinicians not willing to use EHR system  • Financial: initial cost of implementation was high, and funds mainly came from the funding partner  • Functionality: lengthy ANC and immunization e-forms  • Technical: poor internet connectivity as well as software compatibility issues  • Training: no dedicated technical support  • Sustainability: main EHR champion was transferred to another PHC and funding stopped | • Inadequate training because of limited time  • Staff issues relating to the availability of skilled personnel; for example, IT support  • Incomplete evaluation  • Power outages  • Poor network connectivity  • Damaged equipment  • EHR system could not fully replace paper system  • Using both paper and electronic records  • Cost of implementation | • System (infrastructure): poor electricity, inadequate hardware, equipment theft, and networking issues  • People: low levels of computer literacy, reported high user workload, limited support staff, and lack of user buy-in, as well as training barriers resulting from varying staff schedules  • Process: commissioning of a major project resulted in a shift of attention and resources  • Products: request for additional functionality; comprehensive testing needed to ascertain whether all changes requested were captured |

^a^PHC: primary health center.

^b^ToC: theory of change.

^c^MCH: maternal and child health care.

^d^OpenMRS: Open Medical Records System.

^e^ANC: antenatal care.
